# KRAS mutation in colorectal cancer metastases after adjuvant FOLFOX for the primary

**DOI:** 10.1038/bjc.2012.419

**Published:** 2012-09-27

**Authors:** J-N Vauthey, S Kopetz, T A Aloia, A Andreou

**Affiliations:** 1 Department of Surgical Oncology, The University of Texas MD Anderson Cancer Center, 1515 Holcombe Boulevard, Unit 1484, Houston, TX 77030, USA; 2 Department of Gastrointestinal Medical Oncology, The University of Texas MD Anderson Cancer Center, 1515 Holcombe Boulevard, Unit 426, Houston, TX 77030, USA

**Sir,**

We read the article by Kawamoto *et al* (2012) entitled ‘KRAS mutations in primary tumours and post-FOLFOX metastatic lesions in cases of colorectal cancer’ with great interest. This study examined *KRAS*, *NRAS*, *BRAF* and *PIK3CA* mutations in 21 patients who were treated with FOLFOX as adjuvant therapy for stage III/IV colorectal cancer following curative resection. The authors showed that the mutational status of *KRAS* remained concordant between the primary tumours and the post-FOLFOX metastatic lesions, irrespective of patient background, treatment duration and disease-free survival (DFS). This study also suggested that *KRAS* mutation rates were significantly higher in lung than in liver metastases as previously shown (Tie *et al*, 2011).

We congratulate the authors for studying somatic gene mutations after adjuvant treatment of colorectal cancer with FOLFOX. However, an important question that arises from this study is why most patients who recurred after FOLFOX treatment had mutations (17 out of 21; 81%), whereas only a small number of patients were noted to have wild-type tumours (4 out of 21; 19%)? This is much lower than would have been expected, based on the 35–40% *KRAS* mutation rates reported in primary tumours (Andreyev *et al*, 1998).

We have recently analysed the outcome of patients undergoing resection of metachronous colorectal liver metastases (CLM) after adjuvant chemotherapy with FOLFOX for primary stage III colorectal cancer (Andreou *et al*, 2012). This analysis indicated that patients treated with FOLFOX after resection of colorectal cancer had lower survival and an increased rate of *KRAS* mutations in CLM compared with patients treated with 5-FU only. DFS and overall survival (OS) rates after hepatectomy were worse in patients treated with adjuvant FOLFOX (DFS at 3 years: 14% *vs* 38% (5-FU) (*P*<0.0001), OS at 3 years: 58% *vs* 70% (5-FU) (*P*=0.036)). Mutation analysis of liver resection specimens revealed *KRAS* mutations in 47% of patients after FOLFOX, and only 22% after 5-FU (*P*=0.015). Thus, our study suggested that adjuvant FOLFOX may provide a selection pressure favoring a chemotherapy-resistant subset enriched for KRAS mutations while on balance, preventing liver recurrences of patients with KRAS wild-type primary tumours.

Therefore, the high rate of KRAS mutations after adjuvant FOLFOX therapy in this and in our study suggests that although Kawamoto *et al* (2012) confirm that there is *concordance* of *KRAS mutational status* between primary and metastatic lesions (Oudejans *et al*, 1991; Etienne-Grimaldi *et al*, 2008; Santini *et al*, 2008; Italiano *et al*, 2010; Knijn *et al*, 2011), there might be *discordance* of *KRAS mutational rates* because of the selection of a more aggressive form of metastatic disease as a result of therapy.
